# Orexin receptor 2 agonists: a pathophysiologic approach to narcolepsy type 1

**DOI:** 10.1097/MS9.0000000000004476

**Published:** 2025-12-02

**Authors:** Zainab Arif, Erum Siddiqui, Syeda Fadak Zahra Hujjat, Muhammad Saad Khan, Abubakr Mauhmoud

**Affiliations:** a Department of Medicine, Jinnah Sindh Medical University, Karachi, Pakistan; b Department of Medicine, Allama Iqbal Medical College, Lahore, Pakistan; c Department of Medicine, University of Khartoum, Khartoum State, Sudan

**Keywords:** disease-modifying therapy, excessive daytime sleepiness, firazorexton, narcolepsy type 1, orexin receptor 2 agonists, TAK-861

## Abstract

Narcolepsy type 1 (NT1) is a chronic neurological disorder characterized by excessive daytime sleepiness, cataplexy, and disturbed nocturnal sleep, resulting from the loss of hypothalamic neurons that produce orexin. Current therapies, including stimulants, antidepressants, and sodium oxybate, primarily offer symptomatic relief without addressing the underlying orexin deficiency and often have safety or adherence limitations. Orexin receptor 2 (OX2R) agonists represent a novel therapeutic strategy aimed at restoring physiological wakefulness by directly targeting the core neurochemical deficit. TAK-994, the first oral OX2R agonist, demonstrated remarkable efficacy in reducing cataplexy and improving wakefulness but was discontinued due to dose-dependent hepatotoxicity. TAK-861 (oveporexton), a next-generation OX2R agonist with improved structural properties, has shown encouraging Phase 2 results with comparable efficacy and a favorable safety profile, including minimal hepatic effects and once-daily dosing convenience. If validated in large-scale Phase 3 studies, TAK-861 could become the first disease-modifying therapy for NT1, representing a paradigm shift from symptomatic management to mechanism-based treatment. The ongoing development of OX2R agonists highlights a promising frontier in precision sleep medicine, offering renewed hope for patients by targeting orexin restoration.

## Background

Narcolepsy type 1 (NT1) is a chronic neurological disorder characterized by excessive daytime sleepiness (EDS), cataplexy, sleep paralysis, hypnagogic hallucinations, and disrupted nocturnal sleep^[1]^. Sleep-wake stability is dysregulated as a result of the condition’s selective loss of neurons that produce hypothalamic orexin (hypocretin). Neuropeptides called orexins (orexin-A and orexin-B) are essential for regulating sleep-wake transitions and encouraging wakefulness^[2]^. In contrast to NT2, where orexin insufficiency is not a defining feature, NT1 is characterized by cerebrospinal fluid orexin levels that are either undetectable or drastically decreased, usually ≤110 pg/mL^[3]^. Instead of treating the underlying pathophysiology, most NT1 therapies concentrate on managing symptoms. While stimulants like modafinil, armodafinil, and amphetamines help with EDS, they do not help with cataplexy and can have negative effects on cardiac function or tolerance^[4]^. Although sodium oxybate and its more recent formulation, low-sodium oxybate, help with cataplexy and EDS but they also have the potential to cause addiction, respiratory depression, and sleep difficulties^[5]^. Off-label use of antidepressants (such as venlafaxine and fluoxetine) for cataplexy is common; however, there is little evidence to support its use and it may also result in symptoms of withdrawal.

Significant unmet needs in the management of NT1 persist despite the availability of various medications. Long-term adherence is frequently hampered by side effects, many patients still have poor symptom control, and current medications do not replace defective orexin signaling. However, the development of orexin receptor 2 (OX2R) agonists marks a revolutionary change in treatment, as they may have the potential to treat the underlying cause of NT1 instead of symptoms only^[6]^. This manuscript has been developed in accordance with the TITAN Guidelines 2025^[7]^.

## OX2R agonists: mechanism of action

Orexins mediate their physiological effects through two G-protein-coupled receptors: OX1R, which primarily modulates reward processing, stress responses, and autonomic functions, and OX2R, the receptor critically responsible for promoting wakefulness and suppressing REM sleep^[8]^. OX2R agonists are the focused therapy approach intended to pharmacologically compensate for orexin deficit, which is the hallmark pathology in NT1^[9]^. OX2R agonists provide precision activation of the endogenous orexin pathway, potentially restoring natural sleep-wake architecture instead of just relieving symptoms, in contrast to traditional stimulants that non-specifically boost monoaminergic signaling^[10]^.

Two investigational OX2R agonists have gained attention. These agents represent a first-in-class mechanism that goes beyond symptomatic alleviation to precision-targeted therapy opening the door for a time when narcolepsy’s underlying cause can be directly addressed improving patients’ total quality of life in addition to wakefulness:

- TAK-994: A first-in-class oral OX2R agonist that demonstrated strong efficacy in Phase 2 trials but was discontinued due to hepatotoxicity^[11]^.
- TAK-86 (oveporexton): A next-generation OX2R agonist with structural modifications to improve safety while maintaining efficacy^[12]^.

## Clinical trial insights: TAK-994 and TAK-861

### TAK-994 Phase 2 trial (NEJM, 2023)

A Phase 2 trial of TAK-994, which was published in the *New England Journal of Medicine* in 2023, provided the first clinical data in favor of OX2R agonists for NT1^[11]^. Cataplexy was almost entirely eradicated in the majority of patients, and excessive daytime drowsiness (as determined by the Epworth Drowsiness Scale and Maintenance of Wakefulness Test) was significantly reduced in this randomized, double-blind, placebo-controlled study. A paradigm change from symptom management to disease-modifying therapy was signaled by the drug’s capacity to restore natural sleep-wake architecture. However, dose-dependent hepatotoxicity and other side effects, such as weight loss, tachycardia, and urgency in the urine, led to the trial’s termination. Although TAK-994’s development was put on hold, its effectiveness in reducing symptoms confirmed the OX2R agonist strategy and opened the door for compounds with better safety profiles in the future. Targeting the orexin pathway in NT1 treatment has both revolutionary potential and developmental challenges, as demonstrated by this seminal study^[11]^.

### TAK-861 Phase 2 trial (NEJM Evidence, 2024)

Building on lessons from TAK-994, the structurally refined OX2R agonist TAK-861 was developed to maintain therapeutic efficacy while addressing hepatotoxicity concerns. Results from early Phase 2 trials show encouraging results: similar reductions in EDS and cataplexy are achieved by TAK-861 compared to its predecessor, and preliminary safety data indicate no substantial liver toxicity, which is a considerable improvement^[12]^. Furthermore, compared to existing treatments like sodium oxybate, which must be used twice nightly, its potential for once-daily dosing may provide a significant advantage. Although bigger Phase 3 trials are needed to prove TAK-861’s eventual viability, these results position it as a top next-generation OX2R agonist. This development highlights the ongoing potential of tailored orexin receptor agonism to revolutionize the treatment of NT1^[12]^. In these clinical studies, TAK-994 (firazorexton) was administered orally at doses of 30–60 mg twice daily for up to 8 weeks, whereas TAK-861 (oveporexton) demonstrated efficacy at oral doses of 10–50 mg once daily with treatment durations extending to 6–8 weeks in Phase 2 trials.

## Safety challenges

The main side effects of TAK-994 were fatigue, nausea, and reversible hepatotoxicity due to dose-dependent increases in liver transaminases. With the majority of side effects being mild to moderate (headache, sleeplessness, and temporarily elevated heart rate), TAK-861 has demonstrated a more favorable safety profile. However, research on long-term hepatic and autonomic safety data is still ongoing, so cautious optimism is warranted. The termination of TAK-994’s development highlights a number of significant obstacles in the development of OX2R agonists for the treatment of narcolepsy. The most important of them is the unresolved problem of hepatotoxicity, whose exact mechanism is yet unknown but may entail hazardous metabolite accumulation or off-target effects. Targeted therapy is further complicated by autonomic side effects, such as elevated heart rate and urine symptoms, which emphasize OX2R’s wider physiological functions beyond sleep-wake regulation. These results highlight the need for a careful balance in dose optimization, where the hazardous overstimulation of the body must be carefully considered in order to achieve therapeutic efficacy. Moreover, both TAK-994 and TAK-861 trials had limitations, including small sample sizes, short duration, and exclusion of patients with comorbidities. The long-term efficacy, hepatotoxicity mechanisms, and potential for tolerance development remain to be fully elucidated in larger multicenter studies. All of these difficulties show how difficult it is to create OX2R agonists while maintaining patient safety, which calls for more study to improve this promising treatment strategy.

### Comparison to current treatments

Table 1 represents the comparison of OX2R agonist with other narcolepsy treatments such as modafinil and sodium oxybate. OX2R agonists could surpass current therapies if their safety is confirmed while offering targeted, disease-modifying effects (Table 1).

**Table 1:** Comparison of OX2R agonist with other therapies

Feature	Stimulants (modafinil)	Sodium oxybate	OX2R agonists (TAK-861)
Mechanism	Dopamine/norepinephrine modulation	GABA-B agonist	Direct OX2R activation
EDS improvement	Moderate	Strong	Strong (potentially better)
Cataplexy effect	None	Strong	Strong (near-complete)
Safety	Cardiovascular risk	Abuse potential, respiratory depression	Hepatotoxicity (TAK-994), but TAK-861 appears safer
Dosing	Once/twice daily	Twice nightly	Once daily (expected)

## Clinical potential and future implications

If successful, TAK-861 would stand as the first therapy to restore the lost orexin signaling at the core of NT1, marking a genuine shift from symptomatic relief toward true disease modification and thereby truly fulfilling the criteria for a disease-modifying therapy. TAK-861 could transform the treatment of NT1 by being the first medication to specifically target orexin insufficiency, the disorder’s primary etiology, if Phase 3 trials are successful. Through stabilizing the sleep-wake circuitry and repairing impaired OX2R signaling, TAK-861 may lessen dependency on existing polypharmacy regimens (such as stimulants and sodium oxybate) and enhance long-term results. Future studies should concentrate on three main areas: first, determining hepatotoxicity-related biomarkers to inform tailored treatment; secondly, investigating combination therapies, like OX2R agonists and sodium oxybate, for cases that are resistant; and lastly, researching potential uses in NT2 and other hypersomnias, where targeted modulation of dysfunctional, but not absent, orexin signaling may still be beneficial. The success of OX2R agonists may also extend beyond NT1 with potential implications for idiopathic hypersomnia, circadian rhythm disorders, and neuropsychiatric illnesses where sleep-wake dysregulation is common. Future directions may include exploring combination therapies (OX2R agonist with sodium oxybate or stimulants), development of longer-acting formulations, and identification of patient subgroups most likely to benefit from personalized dosing strategies.

## Conclusion

In the therapy of NT1, OX2R agonists mark a paradigm change from symptomatic relief to addressing the root cause. Despite the hepatotoxicity of TAK-994, there is still hope because of TAK-861’s encouraging safety and efficacy profile. In the near future, these drugs may change NT1 therapy; thus, neurology and sleep medicine clinicians should keep an eye on incoming Phase 3 results. The development of orexin-based treatments from the lab to clinical use highlights both the great potential and the challenges of precision medicine in managing sleep disorders. As we await further developments, one thing is clear that the future of narcolepsy treatment lies in restoring what was lost, i.e., orexin signaling.

## Data Availability

No data were generated for this manuscript.

## Research registration unique identifying number (UIN)

Name of the registry: Not applicable. Unique identifying number or registration ID: Not applicable. Hyperlink to your specific registration (must be publicly accessible and will be checked): Not applicable.
